# Cholesterol content in cell membrane maintains surface levels of ErbB2 and confers a therapeutic vulnerability in ErbB2-positive breast cancer

**DOI:** 10.1186/s12964-019-0328-4

**Published:** 2019-02-20

**Authors:** Jinrui Zhang, Qiong Li, Yueguang Wu, Duchuang Wang, Lu Xu, Yang Zhang, Shanshan Wang, Taishu Wang, Fang Liu, Mohamed Y. Zaky, Shuai Hou, Shuyan Liu, Kun Zou, Haixin Lei, Lijuan Zou, Yingqiu Zhang, Han Liu

**Affiliations:** 1 Institute of Cancer Stem Cell, Dalian Medical University, Dalian, China; 2 The Second Affiliated Hospital, Dalian Medical University, Dalian, China; 3 Molecular Physiology Division, Department of Zoology, Faculty of Science, Beni-Suef University, Beni Suef, Egypt; 4 Department of Radiotherapy Oncology, the First Affiliated Hospital of Dalian Medical University, Dalian, China; 5 Cancer Biotherapy & Translational Medicine Center of Liaoning Province, Dalian Medical University, Dalian, China

**Keywords:** ErbB2, Breast cancer, Cholesterol, Membrane fluidity, Membrane rigidity, Lovastatin

## Abstract

**Background:**

ErbB2 overexpression identifies a subset of breast cancer as ErbB2-positive and is frequently associated with poor clinical outcomes. As a membrane-embedded receptor tyrosine kinase, cell surface levels of ErbB2 are regulated dynamically by membrane physical properties. The present study aims to investigate the influence of membrane cholesterol contents on ErbB2 status and cellular responses to its tyrosine kinase inhibitors.

**Methods:**

The cholesterol abundance was examined in ErbB2-positive breast cancer cells using filipin staining. Cellular ErbB2 localizations were investigated by immunofluorescence with altered membrane cholesterol contents. The inhibitory effects of the cholesterol-lowering drug lovastatin were assessed using cell proliferation, apoptosis, immunoblotting and immunofluorescence assays. The synergistic effects of lovastatin with the ErbB2 inhibitor lapatinib were evaluated using an ErbB2-positive breast cancer xenograft mouse model.

**Results:**

Membrane cholesterol contents positively correlated with cell surface distribution of ErbB2 through increasing the rigidity and decreasing the fluidity of cell membranes. Reduction in cholesterol abundance assisted the internalization and degradation of ErbB2. The cholesterol-lowering drug lovastatin significantly potentiated the inhibitory effects of ErbB2 kinase inhibitors, accompanied with enhanced ErbB2 endocytosis. Lovastatin also synergized with lapatinib to strongly suppress the in vivo growth of ErbB2-positive breast cancer xenografts.

**Conclusion:**

The cell surface distribution of ErbB2 was closely regulated by membrane physical properties governed by cholesterol contents. The cholesterol-lowering medications can hence be exploited for potential combinatorial therapies with ErbB2 kinase inhibitors in the clinical treatment of ErbB2-positive breast cancer.

## Background

*ErbB2*, which is also called *Her2* or *Neu*, encodes a receptor tyrosine kinase from the EGFR/ErbB family. Unlike other ErbB members, ErbB2 has no identified cognate ligand, and thus till now is deemed as an orphan receptor [1]. Nevertheless, due to the unique conformation of its extracellular domain, this receptor tyrosine kinase is considered as a favorable dimerization partner among ErbB family members [2, 3]. Amplification of *ErbB2* gene is frequently observed in cancer patients, which identifies a subgroup of breast cancers called Her2/ErbB2-positive that accounts for 20–30% of breast malignancies. *ErbB2* amplification leads to the accumulation of surplus ErbB2 receptors on cell membrane, promoting receptor dimerization and subsequent activation of a wide array of downstream oncogenic signaling circuitries [4, 5]. Hence, the overexpression of ErbB2 inversely correlates with patient prognosis, while ErbB2 has proved as a top therapeutic target in breast cancer treatment with multiple ErbB2-targeted therapies received FDA approvals [6–8].

ErbB2 is a single pass transmembrane receptor embedded in the plasma membrane, a complex structure composed of primarily lipids and proteins [9–11]. Among its many essential physiological functions, cell membrane plays an important role to maintain the homeodynamics of cell surface proteins including the receptor tyrosine kinase ErbB2 [12–14]. On average, about half of the weight of eukaryotic plasma membranes can be attributed to lipids, which form the bilayer membrane structures incorporating three types of amphipathic lipids: phospholipids, sterols, and glycolipids [15, 16]. The majority of the lipid bilayer is composed of phospholipids and sterols, while glycolipids only make up a small fraction of less than 5% in general.

Cholesterol is the major sterol component of animal cell membranes, which makes up about 30% of the lipid bilayer on average. Acting as essential building blocks of the plasma membranes, cholesterol plays pivotal roles in maintaining the structural integrity and regulating the fluidity of cell membranes [17–20], therefore contributing to the homeodynamics of various membrane proteins on the cell surface. For example, alterations in membrane microviscosity and lipid fluidity mediated by cholesterol depletion or enrichment were revealed to significantly affect the cell surface distribution of membrane proteins in human erythrocytes [21, 22]. Furthermore, regarding its cell membrane-associated functions, cholesterol is also implicated in the modulation of cellular signal transmission and intracellular trafficking through contributing to lipid raft assembly and assisting the formation of endocytic pits [23, 24]. Although the oncogenic properties of ErbB2 in breast cancer has been extensively investigated, the connection between its expression levels and the physical properties of breast cancer cell membranes is obscure. Several proteins including HSP90, flotillin, and caveolin have been shown to regulate the cell surface distribution of ErbB2, but how cholesterol content in cell membrane regulates the overall surface presence of this cancer-driving receptor tyrosine kinase remains elusive so far [25–28]. In the present study, we report that cholesterol content modulates the rigidity and fluidity of plasma membranes to maintain the surface levels of ErbB2 in breast cancer cells, while the reduction in cholesterol abundance in plasma membrane facilitates the endocytic degradation of ErbB2 and thus synergizes with the tyrosine kinase inhibitors against ErbB2 to suppress ErbB2-positive breast cancer growth.

## Methods

### Cell lines

Breast cancer SKBR3, AU565, and HCC1954 cell lines were purchased from the American Type Culture Collection (ATCC). SKBR3 cells were cultured with McCoy’s 5A, while AU565 and HCC1954 cells were cultured with RPMI-1640 media, both supplemented with fetal bovine serum (10%, ExCell Bio, Shanghai) and antibiotics (1% penicillin/streptomycin, Gibco). Cells were maintained in a humidified atmosphere in the incubator (Thermo) at 37 °C with 5% CO_2_.

### Antibodies and other reagents

Mouse anti-ErbB2 (A-2), anti-ErbB2 (9G6), anti-Vinculin antibodies were purchased from Santa Cruz Biotechnology (CA, USA). Rabbit anti-PARP antibody was purchased from Proteintech (Wuhan, China). Rabbit anti-phospho-Akt (Ser473) antibody was purchased from Cell Signaling Technology. Secondary goat anti-mouse and anti-rabbit, donkey anti-goat antibodies were obtained from LICOR. Neratinib (HKI-272) and lapatinib (GW572016) were purchased from Selleck. Oleic acid (OA) and lovastatin were obtained from MeilunBio (Dalian, China). Filipin was obtained from Sigma.

### Cell lysis and immunoblottings

Cells were lysed with the RIPA buffer (10 mM Tris-HCl pH 7.5, 150 mM NaCl, 1% (*w*/*v*) TritonX-100, 0.1% (w/v) SDS, 1% sodium deoxycholate) supplemented with mammalian protease and phosphatase inhibitor cocktails (Sigma) as described previously [29]. Protein concentrations were determined via BCA protein assays (Takara). In general, 20–30 μg of protein samples were loaded per lane onto SDS-PAGE gels to be resolved, before transferred to nitrocellulose membranes (Merck Millipore, USA). Membranes were then blocked with 4% non-fat-milk in PBS at room temperature for an hour, followed by incubation with primary antibodies at 4 °C overnight. The blots were washed with PBS for three times before incubation with secondary antibodies. Membranes were finally washed with PBS and detected on a LICOR Odyssey system. Captured images were quantitated using Image Studio software (Version 4.0) as per manufacturer’s instructions.

### MTT assay

Two thousand breast cancer cells were seeded into each well of 96-well plate and incubated overnight. Next day, cells were treated with indicated inhibitors for 24 h, before MTT (3-(4, 5-dimethylthiazol-2-yl)-2,5 diphenyltetrazolium bromide) incubation for another 3 h. DMSO was added to dissolve formazan and absorbance was recorded at 570 nm using a spectrometer (Perkin Elmer, USA).

### Wound healing assay

Cells were seeded onto 6-well plates to form a confluent monolayer. A wound was generated by scratching with a 200 μl pipette tip. Detached cells were cleaned by PBS washes. Mitomycin was added into growth media to a final concentration of 2.5 μg/ml to stop cell proliferation. Images of wound recovery were captured every 24 h using a phase contrast microscope (Leica, DMI4000B).

### Flow cytometry

For apoptosis assays, cultured cells were processed for Annexin V and PI double staining using an apoptosis assay kit (KeyGEN Biotech, China) according to manufacturer’s instructions. The samples were finally analyzed using a benchtop flow cytometer (Accuri C6, BD Biosciences) and acquired data were analyzed using FlowJo software version 7.6.1 (FlowJo, LLC, USA).

### Cholesterol staining

AU565, SKBR3, and HCC1954 cells were seeded onto coverslips placed in 6-well plates with full media. Next day, cells were washed with PBS for three times and fixed with 4% paraformaldehyde for 15 min at room temperature. Following PBS washes, cells were incubated with 50 μg/ml of filipin for 1 h at room temperature, as described previously [30]. Then coverslips were washed and mounted onto a slide using Mowiol (Sigma). Cholesterol staining was examined with ultraviolet excitation using a fluorescence microscope (Olympus, BX63).

### Immunofluorescence

Cultured SKBR3, AU565, and HCC1954 cells were treated as indicated. After PBS washes, cells were fixed with 4% paraformaldehyde for 15 min and then permeabilized with 0.2% Triton X100. Samples were blocked with 2% BSA in PBS for half an hour, before incubation with primary antibodies for 20 min at room temperature. Coverslips were washed with PBS for three times and incubated with Alexa Fluor® 488-conjugated goat anti-mouse secondary antibodies for 20 min at room temperature. Finally coverslips were washed and mounted onto a slide using Mowiol supplemented with DAPI (for visualization of cell nucleus). Antibody staining was observed under a fluorescence microscope (Olympus, BX63).

### Xenograft mouse model

Animal experiments were performed in accordance with the 1996 National Institutes of Health Guide for the Care and use of Laboratory Animals, and the procedures were approved by the Institutional Animal Care and Use Committee of Dalian Medical University. Female nude mice (4–6 weeks, Balb/c background) were obtained from Vital River Laboratories (Beijing, China) and maintained under sterile conditions during the entire experiments. To generate tumor xenografts, cultured HCC1954 cells (6 million/mouse) were implanted subcutaneously into the right flank of each mouse. The sizes of the xenograft tumors were measured using a vernier caliper every 3 days. After the average size of xenografts reached about 150 mm^3^, the mice were randomly divided into 3 groups (5 mice per group) to receive lapatinib, lovastatin and lapatinib, or vehicle control treatment. Lapatinib was dissolved in 2% DMSO, 30% PEG 300, and 5% Tween 80 in distilled water, while lovastatin was dissolved in 30% PEG 400, 0.5% Tween 80, and 5% propylene glycol in distilled water. Lapatinib and lovastatin were administered by daily oral gavage for 17 consecutive days at dosages of 100 mg/kg and 40 mg/kg, respectively. Tumor volumes were calculated according to the following formula: tumor volume (mm^3^) = (length) × (width)^2^ × 0.52. Tumor xenografts were finally excised from the mice after sacrifice, and processed for Western blotting analyses.

### Statistics

To determine statistical significance, experiments were conducted three times with biological repeats. Experimental results were presented as the means ± standard error of the mean (SEM). Statistical differences between experimental groups were examined by performing Student’s t-test in the GraphPad Prism software (version 7), and a *p* value less than 0.05 was considered as statistically significant.

## Results

### Cholesterol content in cell membrane correlates with ErbB2 localization and cell migration

**Fig. 1:**
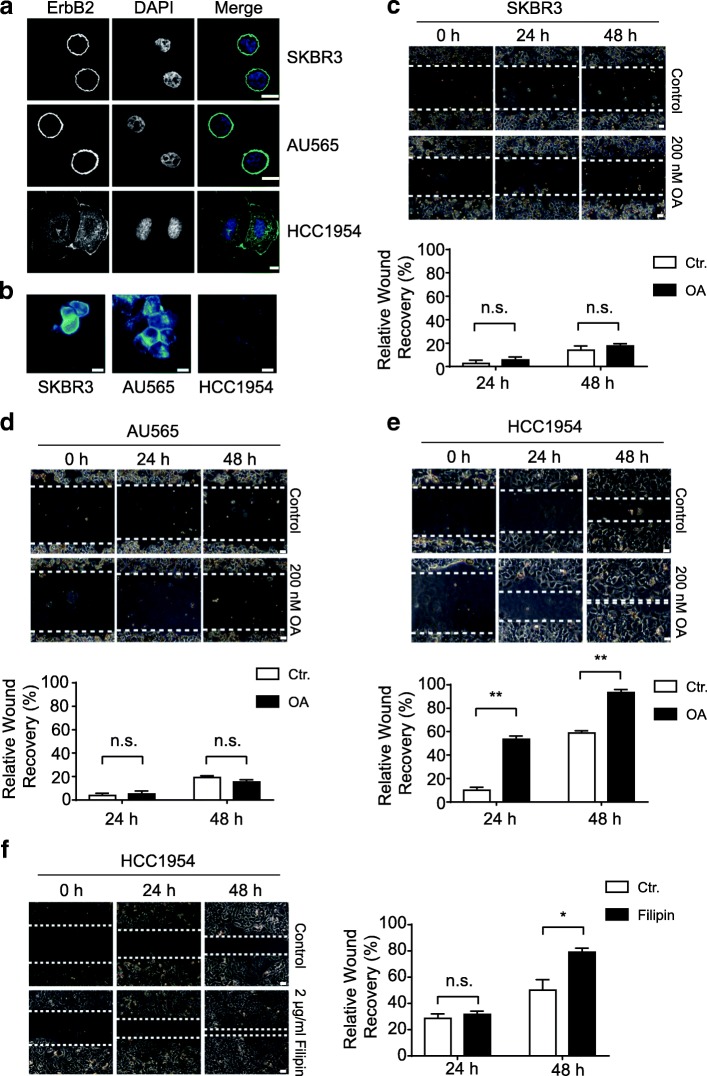
Cell membrane cholesterol content correlates with ErbB2 localization and cell migration in ErbB2-positive breast cancer cells. **a** ErbB2-positive SKBR3, AU565, and HCC1954 cells were cultured on glass coverslips and processed for immunofluorescence analyses with anti-ErbB2 antibody. Nucleus was stained with DAPI. Representative images show confocal sections. Scale bar = 10 μm. **b** indicated cells were cultured and processed for filipin staining to examine cholesterol amounts. Fluorescence images were captured under identical intensity settings. Scale bar = 10 μm. **c**, **d**, **e** and **f**, indicated breast cancer cells were grown to form confluent monolayers and processed for wound healing assays. Mitomycin (2.5 μg/ml) was added during recovery. Cells were either treated with DMSO (control), oleic acid (OA) at 200 nM, or filipin at 2 μg/ml. Images of the wounds were recorded at indicated times under a phase contrast microscope. Scale bar = 50 μm. Adjacent column charts show the quantification of relative wound recovery from each condition. Not significant, n.s., * and ** indicate *p* < 0.05 and *p* < 0.01, respectively

Through immunofluorescence examination of ErbB2 localization in ErbB2-positive breast cancer cells, we observed that, in SKBR3 and AU565 cells that possessed round shapes, ErbB2 was almost exclusively distributed to the cell membrane; while in HCC1954 cells that showed flattened and spread-out configurations, ErbB2 also formed many intracellular punctae besides surface localizations (Fig. 1a). This influence of cell shapes on ErbB2 distribution prompted us to speculate that the physical properties of cell membranes might play a role in the regulation of subcellular distribution of ErbB2, i.e. the round and more rigid cell surfaces in SKBR3 and AU565 cells tended to maintain ErbB2 in the cell membrane, while the floppy and more fluid cell membrane of HCC1954 facilitated the internalization of this receptor. Considering the essential roles of cholesterol in regulating cell membrane rigidity and fluidity [31, 32], we examined the cholesterol content in cell membranes from these three ErbB2-positive breast cancer cell lines. Results from fluorescence microscopy using the cholesterol-specific stain filipin revealed that the cell membrane cholesterol in SKBR3 and AU565 cells was considerably more abundant than that in HCC1954 cells (Fig. 1b).

As cellular cholesterol levels were shown to regulate metastatic phenotypes of cancer cells by altering the fluidity of cell membranes, we investigated whether the differential cholesterol amounts in ErbB2-positive breast cancer cells correlated with their migratory abilities [33]. Indeed, very limited cell migration was observed in SKBR3 and AU565 cells bearing high cholesterol contents, indicative of increased rigidity and reduced fluidity of cell membranes (Fig. 1c and d). Even in the presence of oleic acid that could increase membrane fluidity to some extent, the migration of SKBR3 and AU565 cells was still restricted, likely owing to the relatively high rigidity and low fluidity of the plasma membranes incurred by the abundance of cholesterol (Fig. 1c and d). On the contrary, in HCC1954 cells that contained relatively low levels of cholesterol, cell migration was readily observable, which was further enhanced by treatments with oleic acid or the cholesterol-interfering drug filipin, suggesting that increased membrane fluidity with reduced cholesterol content in cell membrane assisted cell migration (Fig. 1e and f).

### Cholesterol abundance maintains ErbB2 levels on cell surface

**Fig. 2:**
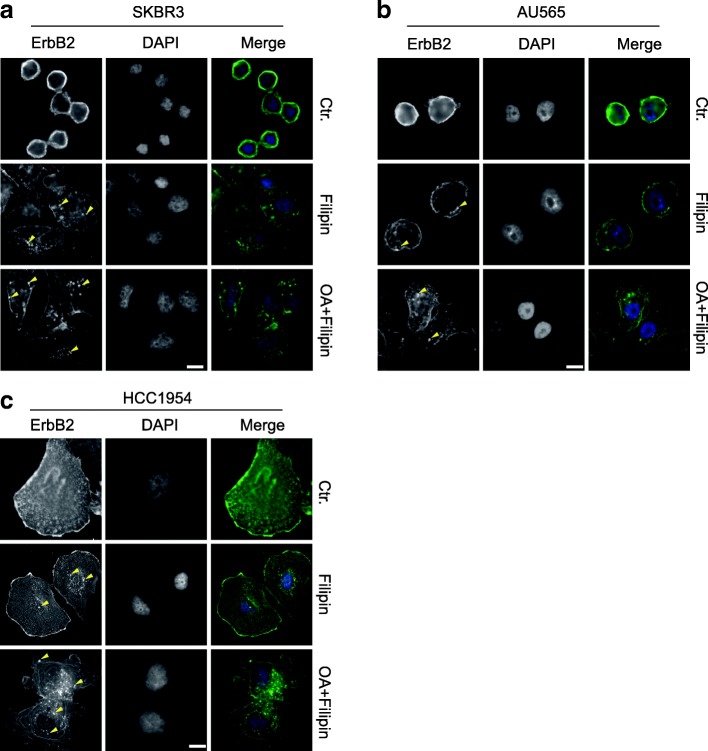
The cholesterol-interfering drug filipin facilitates ErbB2 internalization. **a**, **b** and **c**, ErbB2-positive SKBR3, AU565, and HCC1954 cells were cultured on glass coverslips and treated with filipin (3 μg/ml) with or without oleic acid (OA, 200 nM) for 12 h. ErbB2 location was examined by immunofluorescence. DAPI stains the nucleus. Images were captured using a fluorescent microscope (Olympus BX63, 40X objective). Representative intracellular ErbB2 punctae are indicated with yellow triangles. Scale bar = 10 μm

To further investigate the influence of cholesterol content on the intracellular distribution of ErbB2, we examined the localizations of this receptor in SKBR3, AU565, and HCC1954 cells exposed to the cholesterol-interfering drug filipin. Interestingly, the normally round-shaped SKBR3 and AU565 became more flattened under filipin treatment, and intracellular ErbB2 punctae were readily discernible, suggesting that filipin treatment induced receptor internalization (Fig. 2a and b). In addition, we treated SKBR3 and AU565 cells with the combination of oleic acid and filipin to further increase membrane fluidity and reduce surface rigidity. In this scenario, both SKBR3 and AU565 cells became more spread-out and irregular-shaped, while intracellular ErbB2 staining was greatly enhanced (Fig. 2a and b). On the other hand, although ErbB2 internalization was already evident in HCC1954 cells under normal culturing conditions, the addition of filipin and oleic acid significantly increased the formation of intracellular ErbB2 punctae (Fig. 2c). Taken together, these observations indicate that the cholesterol content in cell membrane regulates membrane rigidity and fluidity to maintain ErbB2 receptors on the cell surface, while the decrease of cholesterol abundance in plasma membrane leads to reduced rigidity and increased fluidity of cell membrane that assist ErbB2 internalization.

### Cholesterol-lowering drug potentiates ErbB2-targeting agents to suppress breast cancer growth

**Fig. 3:**
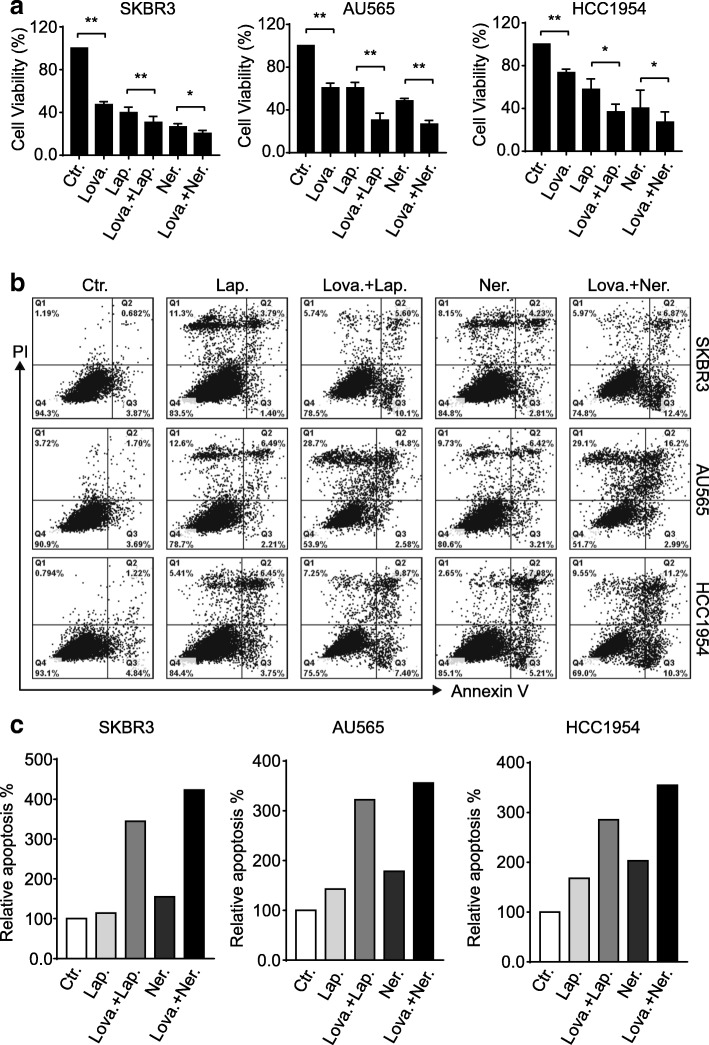
Lovastatin potentiates ErbB2 kinase inhibitors to suppress ErbB2-positive breast cancer cells. **a** SKBR3, AU565, and HCC1954 cells were treated with lovastatin (lova, 40 μM), lapatinib (lap, 200 nM), neratinib (ner, 200 nM), or combinations as indicated for 24 h. Cell viability was then examined via MTT assays. Column charts show data from 3 independent experiments and error bars represent the standard error of the mean (SEM), with * and ** indicating *p* < 0.05 and *p* < 0.01, respectively. **b** breast cancer cells were treated as above and processed for double staining with Annexin V and propidium iodide (PI) to inspect apoptotic populations. **c** column charts show the quantitation of apoptotic cells with positive Annexin V staining (right two quadrants) from **b**

Having disclosed the correlation between cholesterol content in the cell membrane and the surface levels of ErbB2 receptors, we next sought to investigate the influence of the cholesterol-lowering drug lovastatin on the in vitro growth of ErbB2-positive breast cancer cell lines. In doing this, breast cancer cells overexpressing ErbB2 (SKBR3, AU565, and HCC1954) were treated with lovastatin, the FDA-approved tyrosine kinase inhibitors against ErbB2 (lapatinib and neratinib), or the combinations as indicated (Fig. 3a). Results from cell viability assays revealed that lovastatin significantly inhibited cell growth, although less potent than lapatinib and neratinib, but also potentiated the suppression incurred by these two tyrosine kinase inhibitors (Fig. 3a). Furthermore, flow cytometric data from Annexin V and propidium iodide double staining showed that the addition of lovastatin dramatically enhanced the pro-apoptotic effects of the ErbB2 inhibitors, with increases on averages of 3.03 and 2.74 folds in SKBR3, 2.26 and 1.99 folds in AU565, and 1.7 and 1.75 folds in HCC1954 cells observed for lapatinib and neratinib, respectively (Fig. 3b and c). Therefore, these findings suggest that the cholesterol-lowering drug lovastatin is capable of sensitizing the ErbB2-positive breast cancer cells to both lapatinib and neratinib.

### Lovastatin treatment leads to increased endocytic degradation of ErbB2

**Fig. 4:**
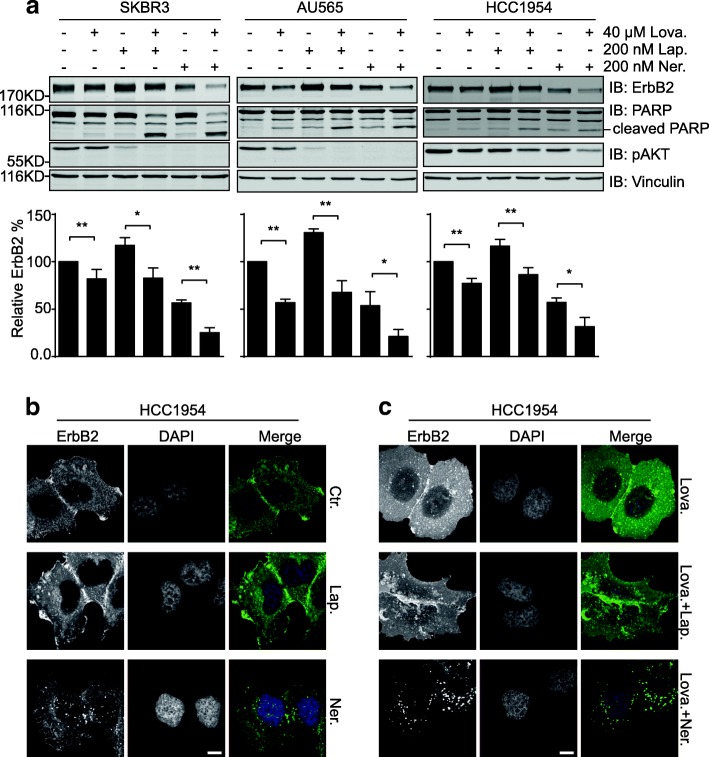
Lovastatin addition results in enhanced internalization and degradation of ErbB2. **a** SKBR3, AU565, and HCC1954 cells were treated with lovastatin (40 μM), lapatinib (200 nM), neratinib (200 nM), or combinations as indicated for 24 h. Cell lysates were analyzed by immunoblotting with indicated antibodies. Column graphs below show the quantification of relative ErbB2 abundance compared to control samples. All error bars represent the standard error of the mean (*n* = 3), with * and ** indicating *p* < 0.05 and *p* < 0.01, respectively. **b** HCC1954 cells were treated with lapatinib (200 nM) or neratinib (200 nM) before immunofluorescence analysis to inspect ErbB2 staining. DAPI stains the nucleus. Scale bar = 10 μm. Images were captured using a fluorescent microscope (Olympus BX63, 40X objective). **c** cultured HCC1954 cells were treated with lovastatin (40 μM) either alone or in the presence of lapatinib or neratinib (both at 200 nM). Immunofluorescence assays were then carried out as in **b**

Given that cholesterol abundance in the cell membrane could maintain the surface levels of ErbB2, we speculated that the inhibitory effects of the cholesterol-lowering drug lovastatin against ErbB2-positive breast cancer cells might be associated with compromised cell membrane expression of ErbB2 and the resultant attenuation of downstream signal transmission. As expected, results from immunoblotting assays revealed that lovastatin treatment led to reduced ErbB2 expression in serum-starved SKBR3, AU565, and HCC1954 cells (Fig. 4a). In accordance with our previous findings, treatments with the ErbB2 inhibitors lapatinib and neratinib resulted in increased and decreased cellular ErbB2 levels, respectively [34]. Nevertheless, lovastatin addition caused significant reductions in ErbB2 expression under the combined conditions compared to inhibitor single treatments, with concomitantly enhanced PARP cleavage and decreased activation of AKT (Fig. 4a). Consistently, in the subsequent immunofluorescence assays to examine the cellular localizations of ErbB2, elevated intracellular ErbB2 staining was readily detected in HCC1954 cells treated with lovastatin (Fig. 4b and c). Even in neratinib-treated cells where ErbB2 endocytosis was induced, the combined treatment with lovastatin further augmented the formation of intracellular ErbB2 punctae (Fig. 4b and c). These immunoblotting and immunofluorescence data collectively suggest that lovastatin facilitated the internalization and intracellular degradation of ErbB2, leading to reduced ErbB2 expression levels and repressed downstream oncogenic signaling.

### Lovastatin synergizes with lapatinib to suppress the in vivo growth of ErbB2-positive breast cancer

**Fig. 5:**
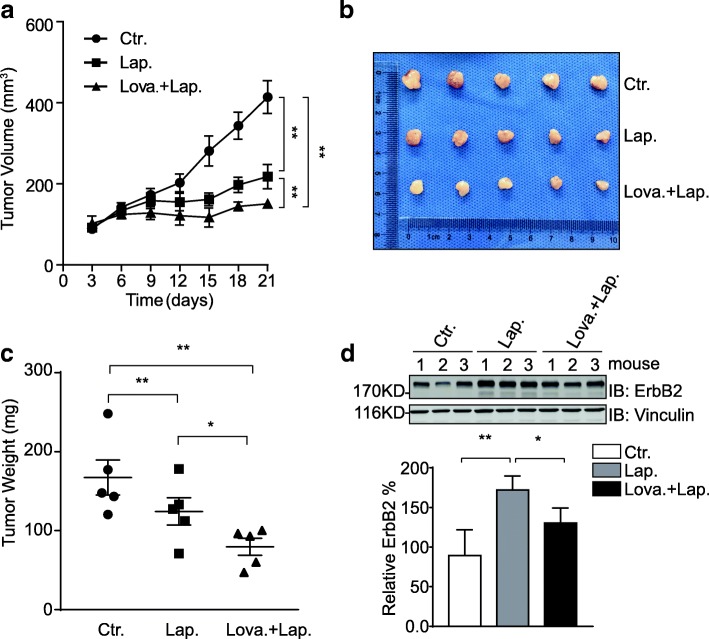
Lovastatin synergizes with lapatinib to suppress HCC1954 xenograft growth. **a** HCC1954 cells were inoculated into female nude mice to generate xenograft mouse models. Mice were randomized into 3 groups to receive control vehicle, lapatinib, or the combination of lovastatin and lapatinib as described in the methods section. The sizes of xenografts were measured every 3 days and calculated tumor volumes were plotted. Presented graph shows data from 5 mice per group. Error bars show the standard error of the mean (SEM), with ** indicating *p* < 0.01. **b** after 17 days of oral gavage treatment, the mice were sacrificed and xenograft tumors were resected. **c** tumor weight was measured and plotted. Presented graph shows data from 5 mice per group. Error bars show the standard error of the mean (SEM), with * and ** indicating *p* < 0.05 and *p* < 0.01, respectively. **d** protein samples were prepared from xenograft tumor tissues (3 per treatment group) and analyzed by immunoblotting using indicated antibodies. Vinculin was probed to show equal loading. Bar chart below shows the relative quantification of ErbB2

Although nearly all types of animal cells generate cholesterol, the majority (80%) of cholesterol synthesis at the organism level is accomplished in the liver and intestines. To investigate the synergistic effects of ErbB2 inhibitor and cholesterol-lowering drug in vivo, we generated ErbB2-positive breast cancer xenograft mouse models by implanting HCC1954 cells into athymic nude mice. Considering that lapatinib is more widely administered than neratinib in the clinical treatment of ErbB2-positive breast cancer, we treated the xenograft-bearing mice with lapatinib or the combination of lapatinib and lovastatin. As illustrated in Fig. 5a-c, lapatinib significantly suppressed HCC1954 xenograft growth in vivo, while the combo treatment with lapatinib and lovastatin further decreased the sizes and weights of xenograft tumors formed in athymic nude mice, suggesting a synergistic effect of lovastatin with lapatinib to restrain the in vivo growth of ErbB2-positive breast cancer. Subsequently, we examined the expression levels of ErbB2 in xenograft tissues resected from mice with different treatments using immunoblotting assays. Consistent with our data from cell line studies, the ErbB2 levels were observed to be elevated in the samples from lapatinib treatment group compared to those of control group; while the combined treatment with lovastatin and lapatinib impeded the lapatinib-induced upregulation of ErbB2, confirming the negative regulatory effect of lovastatin on ErbB2 expression (Fig. 5d).

## Discussion

Breast cancer occurs with the highest incidence among all women malignancies and accounts for about 30% of newly diagnosed women cancer cases annually in the United States [35]. This leading deadly disease is generally divided into luminal A, luminal B, HER2/ErbB2-positive, and triple-negative breast cancer subtypes according to the expression status of estrogen receptor, progesterone receptor, and HER2/ErbB2 [36]. ErbB2 overexpression in breast cancer is always associated with poor patient prognosis [37, 38], but ErbB2 targeted therapies have significantly improved patient survivals, hence confirming this receptor tyrosine kinase as the pivotal therapeutic target for intervention in the treatment of ErbB2-positive breast cancers.

Through the careful observations of distinct patterns in ErbB2 distribution of ErbB2-positive breast cancer cells, we found a correlation between cell shape and ErbB2 localizations wherein ErbB2 remained mainly on the surface in cells with round shapes but became internalized in those with irregular forms. We speculated that the physical features of cell membranes, including rigidity and fluidity, might play a role to influence the cellular ErbB2 localizations. It was therefore intriguing to observe the remarkable difference in cholesterol contents between SKBR3/AU565 and HCC1954 cells. Cholesterol is a major component of animal cell membranes, which is required to maintain the integrity and regulate the fluidity of plasma membranes [23]. Cholesterol contains a tetracyclic ring structure and adopts a *trans* conformation, rendering the molecule a planar and rigid characteristics that increases membrane packing and contributes to the integrity and rigidity of cell membranes. Cholesterol is also a key determinant of membrane fluidity: at high temperatures, cholesterol acts to stabilize the cell membrane and increase its melting point; while at low temperatures, it inserts into phospholipids and prevents them from interfering with each other to avoid aggregation [39]. Consistent with our hypothesis, the cholesterol abundance conferred the cell membranes of SKBR3 and AU565 cells increased rigidity and reduced fluidity that collectively limited cell mobility. In addition, by manipulating the cholesterol content in cell membranes using oleic acid and the cholesterol-interfering drug filipin, the cell shapes of SKBR3 and AU565 could be effectively altered and subsequent ErbB2 internalization was apparently enhanced.

These findings inspired us to investigate the therapeutic effects of cholesterol lowering drugs from the statin class of the HMG-CoA reductase inhibitors, such as lovastatin, on the in vivo and in vitro growth of ErbB2-positive breast cancers. Through a series of cell phenotypic experiments including cell viability and apoptosis assays, we demonstrated that lovastatin significantly potentiated the inhibitory effects of the FDA-approved ErbB2 kinase inhibitors, lapatinib and neratinib. From the mechanistic aspect, we revealed that lovastatin addition upon lapatinib and neratinib treatment led to reduced cellular ErbB2 levels compared to inhibitor monotherapies, likely due to elevated internalization and degradation of ErbB2 incurred by altered membrane physical properties. More importantly, using ErbB2-positive breast cancer xenograft mouse models, we confirmed the potent synergistic effects of lovastatin and lapatinib to suppress the in vivo growth of HCC1954 xenografts.

**Fig. 6:**
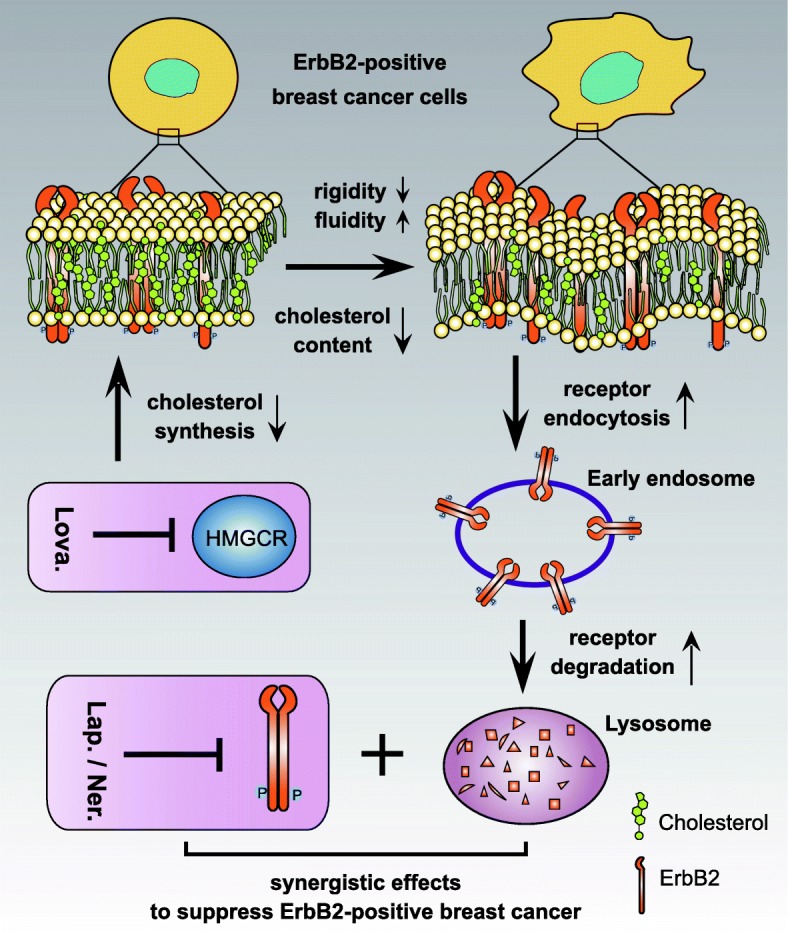
Schematic diagram depicting the synergistic effects of lovastatin with lapatinib. Through inhibiting HMGCR activity, lovastatin reduces cellular cholesterol contents and hence alters the physical properties of the cell membrane, conferring increased fluidity but decreased rigidity. These changes facilitates the internalization of cell surface ErbB2, which leads to its intracellular degradation through endosome to lysosome sorting. Reduced cellular ErbB2 expression sensitizes ErbB2-positive breast cancer cells to ErbB2 kinase inhibitors exemplified by lapatinib and neratinib, which collectively elicit more potent anti-cancer effects

It is noteworthy that several statins that received FDA approvals as lipid-lowering medications have been reported to suppress breast cancer growth. For example, simvastatin was shown to induce PTEN transcription by interfering with NFκB activity to inhibit the proliferation of breast cancer cells [40]. This statin was also observed to inhibit the mammosphere formation and migration of triple-negative breast cancer cells through the regulation of FOXO3a [41]. Lovastatin was reported to suppress the tumor growth and metastasis in a mouse breast cancer animal model by causing increased apoptosis and decreased DNA synthesis [42]. Lovastatin was also shown to suppress the glycolytic and citric acid cycle activity to inhibit the proliferation of breast cancer MDAMB231 and MDAMB468 cells [43]. Furthermore, a recent study proposed that lovastatin in a cerasome-encapsulated nanohybrid form effectively suppressed the stemness properties of triple-negative breast cancer [44]. In addition, pitavastatin was recently reported to sensitize breast cancer to radiation by delaying DNA Repair and promoting senescence [45]. Therefore, it seemed that the statin class of medications exhibited pleiotropic effects to suppress the growth of breast cancer, while our findings revealed a novel aspect of the anti-cancer properties of the statins. Lovastatin, and likely other statins, through compromising HMG-CoA reductase activity, reduced cellular cholesterol abundance and led to altered rigidity and fluidity of the cell membranes. The resultant membrane physical properties facilitated the internalization and subsequent degradation of cell surface ErbB2, which synergized with lapatinib or neratinib to elicit potent inhibitory effects on ErbB2-positive breast cancer growth both in vitro and in vivo (Fig. 6).

## Conclusions

ErbB2 has proven as a top therapeutic target in the clinical treatment of breast cancer. In the present study, we report that the cholesterol content in cell membranes regulates the surface levels of ErbB2 via affecting membrane rigidity and fluidity. Cholesterol-lowering drugs exemplified by lovastatin potentiated the inhibitory effects of ErbB2 kinase inhibitors by down regulating ErbB2 expression. Therefore, our findings warrant further investigations to reveal the synergistic effects of the statin class of medications with tyrosine kinase inhibitors against ErbB2 in the clinical management of ErbB2-positive breast cancers.

## Abbreviations

EGFR: Epidermal Growth Factor Receptor
HMGCR: HMG-CoA reductase (3-hydroxy-3-methyl-glutaryl-coenzyme A reductase
Lap: Lapatinib
Lova: Lovastatin
Ner: Neratinib
OA: Oleic Acid
PBS: Phosphate-Buffered Saline
SEM: Standard Error of the Mean
